# Granzyme B PET Imaging of the Innate Immune Response

**DOI:** 10.3390/molecules25133102

**Published:** 2020-07-07

**Authors:** Kathleen M. Capaccione, Mikhail Doubrovin, Nikunj Bhatt, Akiva Mintz, Andrei Molotkov

**Affiliations:** Department of Radiology, Columbia University Irving Medical Center, New York, NY 10032, USA; kmc9020@nyp.org (K.M.C.); md2367@cumc.columbia.edu (M.D.); nb2903@cumc.columbia.edu (N.B.); am4754@cumc.columbia.edu (A.M.)

**Keywords:** inflammation, PET imaging, granzyme Bhr

## Abstract

The human immune system is a complex system which protects against invaders and maintains tissue homeostasis. It is broadly divided into the innate and adaptive branches. Granzyme B is serine protease that plays an important role in both and can serve as a biomarker for cellular activation. Because of this, a granzyme B PET agent (GZP) has recently been developed and has been shown to be able to monitor response to immunotherapy. Here, we evaluated the utility of granzyme B PET imaging to assess the innate immune response. We subcutaneously administered LPS to mice to induce inflammation and performed granzyme B PET imaging after 24 and 120 h. We dissected out tissue in the region of injection and performed granzyme B immunofluorescence (IF) to confirm specificity of the GZP radiotracer. Granzyme B PET imaging demonstrated increased uptake in the region of LPS injection after 24 h, which normalized at 120 h. Granzyme B immunofluorescence showed specific staining in tissue from the 24 h time point compared to the PBS-injected control. These findings support the use of granzyme B PET for imaging innate immunity. In certain clinical contexts, the use of GZP PET imaging may be superior to currently available agents, and we therefore suggest further preclinical studies with the ultimate goal of translation to clinical use.

## 1. Introduction

The human immune system is a complex and dynamic system comprised of cells and signaling molecules that orchestrate the body’s response to invaders of many different types [1]. It plays critical roles in the development and maintenance of tissue homeostasis and cellular cross-talk with other types of cells [2]. Importantly, it performs surveillance of the body’s own cells to eliminate any developing genetic aberrations that would result in dysplasia and ultimately cancer [3,4].

The immune system is divided into the innate and adaptive branches which respond to different cellular assaults and employ specific cell types [2,5]. Recent advances in immunology have proven these to be complex and interwoven biological responses leading to a cascade of changes within an organism [6]. The innate immune system responds first to invaders, rapidly orchestrating the body’s response. Epitopes on invading pathogens and at sites of tissue damage signal to natural killer cells, macrophages, eosinophils, neutrophils, and dendritic cells to extravasate from the circulation where these cells mark them for destruction through the complement cascade [7]. Granzyme B is a serine protease that functions in conjunction with perforin in pore formation in the membranes of cells marked for destruction [8,9]. Several mechanisms by which granzyme causes apoptotic target cell death have been defined, including cleavage of target cell proteins resulting in apoptosis and interference with cell replication machinery [8,10,11]. For this reason, increased granzyme B can serve as a biomarker for the innate immune response [12,13].

Recently granzyme B has been extensively studied for its role in the adaptive immune response in the context of cancer immunotherapy [14,15]. Granzyme B plays a role in one of two major pathways by which T-cells mediate cell death, making it an important marker of T-cell activation [16,17]. Based on the important role of granzyme B, a novel PET imaging agent was developed as a predictive biomarker for response to immunotherapy [18,19,20]. Larimer et al. first established in a syngeneic murine model of colon adenocarcinoma that the elevation of granzyme B seen in immunoblot and immunohistochemical studies was demonstrated on PET imaging using the novel granzyme B PET (GZP) agent that they developed [18]. This group went on to demonstrate that a response to immunotherapy could be imaged using this PET agent [19]. These promising results suggest the GZP may be a useful clinical PET tracer for monitoring progression of disease in patients on immunotherapy.

Here, we utilized the novel granzyme B PET agent described above to assess the innate immune system by inducing inflammation. We hypothesized that it would demonstrate dynamic changes in natural-killer cell mediated granzyme B activity.

## 2. Results

### 2.1. [^68^Ga]Ga-NOTA-GZP PET Imaging

**Figure 1:**
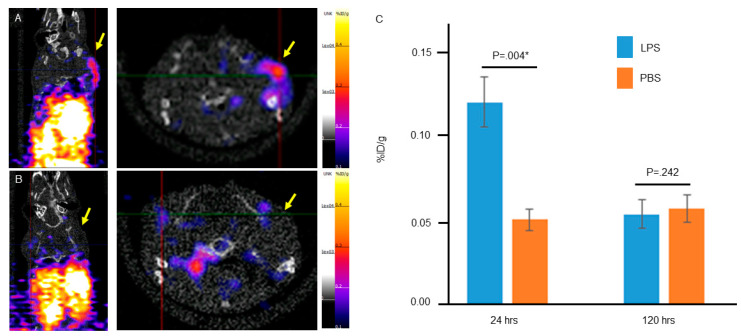
Static granzyme B PET imaging with [^68^Ga]-GZP one hour after radiotracer injection (**A**) 24 h and (**B**) 120 h after injection of LPS. These images show increased uptake in the region of LPS injection at 24 h which resolves at 120 h. (**C**) Quantification of the percent injected dose per gram (%ID/g) demonstrates a statistically significant difference between the LPS (right) and PBS (left) injected shoulders at 24 h, but not at 120 h. * represents statistically significant result, *p* ≤ 0.05.

To assess if the GZP radiotracer could localize specifically to inflammation mediated by innate immunity, we induced inflammation in mice using LPS and performed granzyme B PET imaging. Mice were treated with LPS and then injected with radiotracer 24 h later to allow for induction of a robust innate immune response. Figure 1A demonstrates specific uptake one hour post radiotracer administration in the posterior right shoulder, the region of LPS injection. There was only background uptake in the contralateral PBS injected shoulder.

To validate that this response was the result of LPS-mediated innate inflammation, we again injected mice with LPS however waited 120 h before imaging. Although variable based on cell type and context, it is generally accepted that acute inflammation resolves within 5 days, therefore we hypothesized that we would not see increased GZP uptake at 120 h post LPS injection. Consistent with this hypothesis, GZP PET imaging 120 h post LPS injection did not show substantial increase in uptake at the site of LPS injection (Figure 1B).

Quantification of the PET uptake studies (Figure 1C) were consistent with qualitative analysis of the PET images demonstrating a statistically significant difference in percent injected dose per gram (%ID/g) between the LPS and PBS injected regions at 24 h (*p* = 0.004). Quantification of uptake in LPS and PBS regions 120 h post LPS treatment demonstrated no significant difference in %ID/g. Together, these finding support the specificity of GZP to LPS-induced innate inflammation. To confirm that GZP uptake at the LPS injected site is due to innate immune system response, we tested GZP uptake in T-cell deficient CrTac: NCr-Foxn1nu mice 78 h after LPS injection (Supplementary Figure S1). We demonstrated high uptake at the site of LPS injection in these mice, further supporting that the response is due to the innate immune system.

### 2.2. Granzyme Immunofluorescence

**Figure 2:**
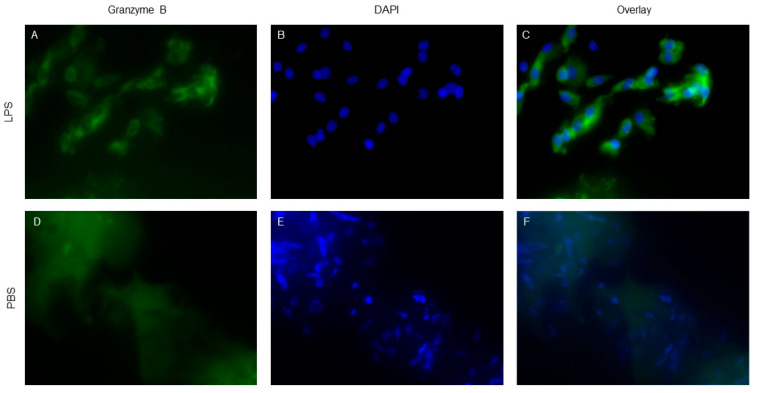
Anti-granzyme B immunofluorescence (IF) in samples 24 h post injection of LPS (**A**–**C**) and PBS (**D**–**F**), respectively. Staining shows specific Granzyme B localization within the cytoplasm of LPS treated tissue compared to the non-specific staining seen in PBS treated control tissue. IF staining closely mirrors PET %ID/g data, supporting specific localization of [^68^Ga]-GZP in cells treated with LPS to induce innate immune response.

To confirm that [^68^Ga]-GZP PET imaging was specific to LPS-induced innate inflammation, animals were sacrificed and tissue in the region of LPS or PBS injections was extracted for further analysis. We performed anti-granzyme B IF on LPS (Figure 2A,C) and PBS (Figure 2C,E) treated tissue which demonstrated differential staining of granzyme B in LPS-treated compared to PBS-treated mice, similar to prior studies [18,21]. These data validate our in vivo findings demonstrating specificity of [^68^Ga]-GZP in LPS-induced inflammation.

## 3. Discussion

Granzyme B imaging using the novel PET tracer GZP has provided a promising avenue for imaging the adaptive immune response in cancer patients treated with immunotherapy [18,19]. Here, we demonstrate the utility of this radiotracer to image innate inflammation. [^68^Ga]-GZP PET imaging of LPS-induced inflammation demonstrated a specific response which was corroborated with tissue staining post-mortem. [^18^F]-FDG-PET imaging has been extensively evaluated for inflammation imaging [22,23] and numerous other agents have been investigated [24,25,26], however none have achieved widespread use. Our preclinical work suggests that granzyme B PET imaging has the potential to be a clinically useful agent for inflammation imaging.

A limitation of [^68^Ga]-GZP imaging demonstrated in this study was hepatic and renal metabolism resulting in high uptake in the liver and kidneys, which would interfere with identification of a site of inflammation in this region. Because granzyme B is also found in the CD8+ T cells of the adaptive immune system, GZP radiotracer would also localize to sites of adaptive immune-mediated infection, limiting specificity. Further dynamic studies are needed to assess how closely GZP B imaging mirrors dynamic cellular change in inflammation before clinical translation.

Despite these limitations, these data are a promising foundation for further studies of GZP PET imaging to assess the innate inflammatory response.

## 4. Materials and Methods

### 4.1. Mouse Model of Inflammation

C57BL/6 and CrTac: NCr-Foxn1nu mice were obtained from Taconic Biosciences and maintained by the Columbia Institute for Comparative Medicine under approved IACUC protocol AC-AAAT9470 (approved 9/12/17). LPS injections were performed according to established protocols [27] using 100 uL of 1 mg/mL LPS with 100 uL growth factor reduced Matrigel (Cat# 354230, Corning, NY, USA). LPS was injected into the right posterior shoulder of each mouse; the same volume of PBS was injected into the contralateral shoulder as a control. All animal experiments were conducted according to protocols approved by the Institutional Animal Care and Use Committee of Columbia University Medical Center.

### 4.2. NOTA-GZP Synthesis and ^68^Ga Radiolabeling

NOTA–β-Ala–Gly–Gly–Ile–Glu–Phe–Asp–CHO (NOTA-GZP) was purchased from CPC scientific, Sunnyvale, CA, USA. ^68^Ga was eluted from a ^68^Ge/^68^Ga generator (GalliaPharm 10055-AMGD02, Eckert & Ziegler, Berlin, Germany). Eluted ^68^Ga (10–12 mCi, 0.37–0.44 GBq) was equilibrated to pH 4.0 with 1M Sodium acetate buffer followed by addition of 20 μg NOTA-GZP. Reaction was incubated at 30 °C for 15 min in a thermomixer (600 rpm). The formation of [^68^Ga]Ga-NOTA-GZP was verified by radio-TLC using Varian ITLC-SA strips and 50 mM EDTA (pH 5) as the mobile phase. In this system, free ^68^Ga moved near to solvent front (*R*_f_ = 0.9 to 1.0) whereas [^68^Ga]Ga-NOTA-GZP remain near to origin (*R*_f_ = 0.0 to 0.1). By following this process, NOTA-GZP was radiolabeled quantitatively with >98% radiochemical purity (*n* > 7). Formed [^68^Ga]Ga-NOTA-GZP used as such without further purification. Molar activity (MA) of formed [^68^Ga]-NOTA-GZP was calculated to be 18.522.2 MBq/μg. We obtained 100% radiochemical yield (decay uncorrected).

### 4.3. In Vivo Murine Model of PET Imaging

24 and 120 h after LPS treatment mice were injected with [^68^Ga]-GZP tracer for PET imaging. Animals were injected with 4–5.7 MBq (108–154 mCi) of the [^68^Ga]Ga-NOTA-GZP (also referenced in the text as [^68^Ga]-GZP). 20 min static PET images were acquired 1 h after [^68^Ga]Ga-GZP injection. PET images were reconstructed using the 3D-OSEM algorithm with 3-iterations in 256 × 256 matrix, without attenuation correction (Inveon, Siemens, Munich, Germany), then post processed using VivoQuant software ver. 4 (Invicro, Boston, MA, USA). Regions of interest (ROI) were drawn manually and % injected dose/gram (%ID/g) was calculated using VivoQuant software.

### 4.4. Immunofluorescence of Granzyme B

After PET imaging, animals were sacrificed and tissue in the region of LPS/PBS injections were dissected, placed in 4% paraformaldehyde for overnight incubation, and transferred to 30% sucrose solution overnight. Subsequently they were embedded in O.C.T. medium (Fischer Scientific, Waltham, MA, USA) and frozen overnight at −80 degrees. Samples were sectioned on a cryostat at 5–10-micron sections.

Anti-Granzyme B (Cat #4059, Abcam, Cambridge, MA, USA) immunofluorescence was performed according to a standard protocol [21] using anti-granzyme B primary antibody 1:100 and a secondary goat anti-rabbit antibody 1:1000 (Thermo Fisher, Waltham, MA, USA). Slides were imaged on an Echo Revolve 332 microscope (Echo) at 4×, 10×, and 40× magnification.

## 5. Conclusions

Granzyme B PET imaging has recently been shown to assess early response to immunotherapy via its role in adaptive immunity. Here, we demonstrated the role of [^68^Ga]-GZP in imaging the innate immune response which has important implications for inflammation PET imaging. Future preclinical studies will lay the groundwork for clinical translation of this experimental radiotracer for PET imaging of human inflammation.

## Supplementary Materials

The following are available online, Figure S1. Granzyme B PET imaging of LPS-induced inflammation in T-cell deficient CrTac:NCr-Foxn1nu mice. High uptake of [^68^Ga]-GZP at the site of LPS injection in these mice support that the response is due to the innate immune system.
