# Supplementary material for: Granzyme B PET Imaging of the Innate Immune Response
Source: Molecules. 2020 Jul 7;25(13):3102. doi: 10.3390/molecules25133102 (PMC7411671; doi:10.3390/molecules25133102)
Supplement: Supplementary file 1 [file molecules-25-03102-s001.pdf]

molecules

MDPI

Supplementary Materials

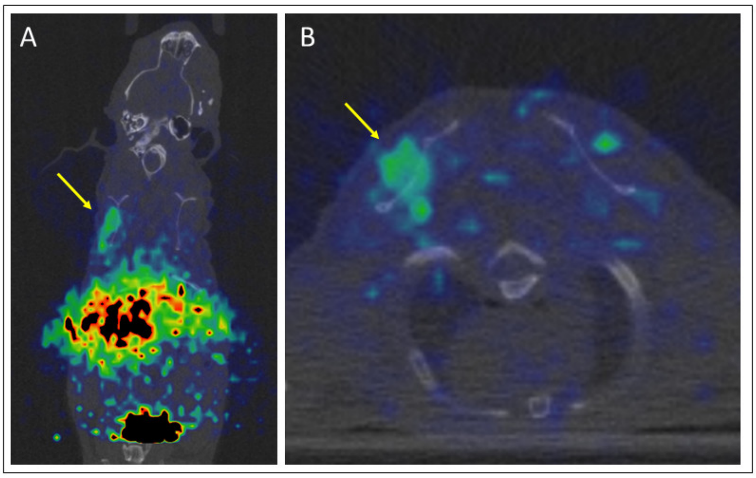

Figure S1. Granzyme B PET imaging of LPS-induced inflammation in T-cell deficient CrTac:NCr-Foxn1nu mice. High uptake of [^{68}Ga]-GZP at the site of LPS injection in these mice support that the response is due to the innate immune system.
